# Medical Topography of Central Arkansas; Being Observations on the Locality, Climate, and Diseases of the City of Little Rock and Vicinity in the Year 1840

**Published:** 1843-05

**Authors:** W. J. Goulding

**Affiliations:** Little Rock


### WESTERN JOURNAL

OF

### MEDICINE AND SURGERY.

---

MAY, 1843.

---

Art. I.—*Medical Topography of Central Arkansas; being Observations on the Locality, Climate, and Diseases of the City of Little Rock and vicinity, in the year* 1840. By W. J. Goulding, M. D., of Little Rock.

The relative importance of Medical Topography to the advancement of our science is now highly appreciated. Many elaborate papers of this kind annually appear in our journals; and we are led to value these successive additions to our medical literature the more from the conviction that, if ever we have a digest worthy to serve as a guide to the practice of medicine in the Southern States, its best materials will be sought in the accumulated contributions of local medical history. Physicians, in innumerable dissertations on our science, have applied to all regions of the earth some aphorisms of Hippocrates applicable only to Greece and a few adjacent countries; this error is now known, and the most accurate observation of Nature in a given climate is found requisite to an enlightened and efficient practice in that climate. Nor is the study of all the physical circumstances of a given district which may affect the health of its inhabitants, alone required; but the peculiarities which the same disease at different times assumes, are especially to be noted; and perhaps no country, from its great extent and varied natural features, calls more imperiously for the careful observation of these facts that the United States. But waiving further preliminaries, I proceed to the more immediate object of this paper.

The city of Little Rock, which represents central Arkansas, is in latitude 34° 40′, and longitude 15° 20′, about 300 miles by water from the mouth of the Arkansas river where it joins the Mississippi. Major Long, who passed it in 1820 says: "It is a village having six or eight houses and occupies a high bank of clay-slate on the south west side of the Arkansas river. Its site is elevated, and the country immediately adjoining is in a great measure exempt from the operation of those causes which produce a state of the atmosphere unfavorable to health." It is in fact the first eligible site for a town that occurs in ascending this river; being near the centre, and the capital of a large and growing state, its future destiny can hardly be mistaken. Besides a United States' Arsenal, it has already many fine public and private buildings. Population, 2,400.

The face of the country of central Arkansas, though much diversified, may be classed under three varieties; first, *alluvial* or river bottom; second, *upland* or hilly; third, *prairie.*

The first variety is chiefly met in descending the river; the soil is of the richest kind; it is the most thickly settled but the least healthy. It is here that the forest is seen in its greatest magnificence, where the cypress, oak and cotton-wood vie in power, lending their support to enormous osier and grape-vines which delight

> "To weave their gorgeous tracery far above;
> With the light melting through their high arcades,
> As through a pillared cloister."

But the want of good water, and the liability of this portion to the annual Nile-like floods of the Arkansas river, weigh against its great natural fertility. Yet the bottom lands of central Arkansas may fairly be set down as excellent of their kind; the banks rarely descend as they recede from the river; corn and cotton are the staple productions.

The second, or upland variety, in the face of the country, is by far the most extensive; it is always rolling, often hilly, with but little undergrowth save luxuriant grasses and flowering herbage; having forest chiefly of oak interspersed with hickory, its whole aspect, so open, park-like, and beautiful, presents a striking contrast to that of the first variety. This second variety constitutes by far the most salubrious district; good springs of water are met with, but are not common; corn and the small grains are grown advantageously, but cotton becomes an uncertain crop; most of the soil is tillable, much of it decidedly good.

The third variety, or prairie, is the least extensive, and is found at a distance east of Little-Rock, between the Arkansas and White rivers; several small praries, and a large portion of "big prairie" so called, are found in this direction; usually of the better kind of dry or upland prairie, they easily admit of cultivation although as yet but little cultivated; their greatest drawback is the want of good water.

From the foregoing it will be perceived that the greatest diversity in the face of the country obtains in central Arkansas; the sea-like prairie and the beetling cliff—the mountain torrent and the sluggish bayou—the sleeping lake and the mighty river, will not unfrequently by quick succession agreeably surprise the traveller or command the fullest admiration of the lover of nature. This marked diversity joined to a genial climate renders it a locality of peculiar interest in regard to its natural productions; the muscadine and other wild grapes, as also the date-plum (*dyospyros pubescens* of Pursh), and the paw-paw (*porcelia triloba*), are found in perfection here. The cane (*miegia*) extends itself high up this river, and the beautiful china-tree is easily grown and is said to have become naturalised here; among the forest trees are found the false orange (*maclura aurantica*), evergreen holly (*ilex opaca*), tupello gum (*liquidambra styraciflua*), and the coffee-bean tree. Among medicinal plants, the *palma christi*, *spigelia*, two species *gillenia* and two *baptisia*, also the more valuable of the natural families *labratae* and *solanae* are here found in the greatest profusion; the tarantula, the scorpion, and centipede, (the last of enormous size) among reptiles; and beautiful specimens of the amethyst and topaz among minerals. Anthracite coal of an excellent quality is at this time an article of export from central Arkansas, and the locality of "Hot-Springs" in this State will yield to none in the world perhaps in geological interest.

I come now to speak more particularly of the climate; in this respect we have a medium between that of New Orleans on the one hand and that of St. Louis on the other; it may also be observed that distance west of the Mississippi, and the more elevated character of the adjacent country renders the air far less humid and the prevailing winds in general more agreeable than in the immediate valley of that river. Ordinarily the peach is in *full* flower quite early in March, and the forest in *full* leaf by the first of April. But as a just estimate of climate can be drawn only from daily and accurate meteorological observations, I will here insert the following table, being an abstract of much more detailed observations for the year 1840, following the same with such other remarks as the farther elucidation of the subject may seem to demand.

It is proper here to observe that, in regard to the position of the instrument, times of noting, &c., in the tables of which the above is an abstract, the indications given on this subject in the "Regulations of the Medical Department of the United

1 *

States Army" have been strictly observed. Range for the year, seventy-seven. Coldest month, January. Hottest month, August. As it respects temperature, the past may be considered an average year in this locality, but in regard to weather it must be stated that the *amount* of *rain* and the proportion of *cloudy* to *fair* has been greater than usual. The prevailing winds also in this locality during the hot season are usually south and south-west; in this respect however the table will show a decided prevalence of east and north-east winds during the months July, August and September, 1840. This fact (which, indeed, was the subject of frequent remark at the time) is believed to have had an important agency in producing the very unusual severity and prevalence of fever, which afflicted this place and vicinity the past season, as will appear more fully in the sequel.

*Diseases.*—The past season as before intimated has been characterised by an unusal prevalence and severity of intermittent and bilious remittent fever. Very early in August these forms of fever assumed a grave character and became unusually prevalent, at the same time an early tendency to general prostration or, in other words, a typhoid diathesis seemed to mark the progress of the disease: emetics early, followed by mild cathartics and mucilaginous drinks, with cold affusions, blisters, and especially later in the attack, the extensive application of mustard, gained an increasing confidence as the disease progressed. It is worthy of remark here that sore-mouths, unusually protracted and obstinate in their character, often followed; nor did these follow only in cases in which mercurials entered the plan of treatment; in very many of these cases the gums would remain in their normal condition while the buccal portions of the mouth and the tongue were the seat of corroding ulcers, tumefaction and intense pain. In these cases Labarraques' liquid was found a most efficient, indeed an invaluable local application. In general the lancet, as also protracted purging, was ill borne, and it soon became manifest that an object of primary importance was to husband the general strength for a sequel of protracted debility, as insidious and dangerous in its character as it was sure to follow. Indeed, when contrasted with the degree of violence attending the invasion and progress of the attack, the period of convalescence was in every case peculiarly protracted and critical. This aggravated prevalence of fever subsided early in October, since which time up to the date of this paper, the health of the locality has been comparatively good. It is not to be inferred that the disease at any time assumed that malignant type; or ran its course with that rapidity which has sometimes characterised the prevalence of epidemic fevers in still more southern and more unfavorably located towns. The interments in the city during the four months ending on the thirty-first day of October, were one hundred and five, of which number eleven were colored persons, and thirty-five were children under ten years of age. Population as before stated two thousand and four hundred. This proportion of mortality to the number of inhabitants is believed to be at least three-fold that of preceding years at the same period. When the commercial location of this town and its rapid growth during the last two years are considered, it will readily be inferred that a large proportion of the deaths were of non-resident or unacclimated persons; this was strikingly the case; indeed comparatively few of those long resident in the locality have fallen victims to the diseases of the season, with which they have suffered in common with others.

In searching for the causes of the peculiar cast and strength of the epidemic influence of this locality the past season, we are as in most cases of the kind not fully satisfied; much we think may be set down to the fact before stated in regard to the winds coming upon us during the hot season from an unusual and unfavorable quarter—and also to the fact that the great annual or June rise in the Arkansas river occurred some four weeks later in the season than is common, and the overflow in central Arkansas was unusually great. The preceding season, to wit, that of 1839, was one of good health, though characterised by uniform and highly sustained heat and protracted drought, so much so that many wells of the town were dry and others unfitted for use; the winter following (that of '39 and '40), was marked by no peculiarities. If we except the season in question, the past history of this place will put it in an enviable rank for health among the towns of the south-west. Yellow-fever has never appeared here, nor has cholera, only as it was imported; generally speaking there are no prevailing diseases save intermittents, which are usually of a mild grade and readily yield to appropriate treatment. Pulmonary consumption can hardly be said to have an existence, at the same time complicated pleurisies (pneumonia biliosa) are not uncommon during our winters, and are the most to be dreaded of any attacks incident to that season; scarlatina occasionally makes its appearance, and instances of chronic rheumatism are not infrequent. Once in the past history of central Arkansas a disease called the "cold-plague" had a brief and limited, but for the time a very fatal existence in Conway county, fifty miles above Little Rock, rapidly carrying off a number of the inhabitants. Two cases (answering to the above disease as it has been described to me), terminating fatally within thirty-six hours, in somewhat aged subjects and of impaired constitutions, have fallen under my observation the past season; these attacks consisted of a violent congestive chill, if I may be allowed the expression, from the almost paralysing effect of which the system was unable to rally.

Next, in regard to prevalence and deletereous agency on health and life in this place, may be mentioned, diarrhœa, dysenteria, croup, cholera infantum, and dropsies. In conclusion it may be remarked that this climate is not subject to as great a variety of diseases as portions of our country farther north. Fever in its multifarious forms far outweighs all others; and it is believed that when the local causes incident to all new districts, tending to aggravate this class of diseases, shall have more fully passed away, this place will be noted for its salubrity as it now is for its beautiful and commanding location.

January 1841.

